# Exploring Factors Influencing Farmers’ Continuance Intention to Crop Residue Retention: Evidence from Rural China

**DOI:** 10.3390/ijerph18147412

**Published:** 2021-07-11

**Authors:** Hao Gai, Tingwu Yan, Anran Zhang, William David Batchelor, Yun Tian

**Affiliations:** 1 College of Economics and Management, Huazhong Agricultural University, Wuhan 430070, China; gaihao0729@163.com (H.G.); zar0428@163.com (A.Z.); 2 Hubei Rural Development Research Center, Wuhan 430070, China; 3 College of Agriculture, Auburn University, Auburn, AL 36832, USA; bbatch@auburn.edu; 4 School of Business and Administration, Zhongnan University of Economics and Law, Wuhan 430073, China; tianyun1986@163.com

**Keywords:** crop residue, residue retention, continuance intention, expectation confirmation model

## Abstract

Over the past decade, crop residue burning after harvest is prevalent in developing countries. Promoting crop residue retention to replace residue burning is effective in improving air quality and contributing to the mitigation of global climate change. This study examines farmers’ continuance intention of crop residue retention, using the expectation confirmation model (ECM) and survey data from 542 rice farmers in Hubei Province, P.R. The results show that farmers’ perceived usefulness, confirmation, perceived ease of use, and satisfaction significantly have direct or indirect positive impacts on their continuance intention on adopting crop residue retention. In particular, perceived ease of use contributes the most. Perceived usefulness and perceived ease of use act as intermediaries in the relationship between confirmation and farmers’ continuance intention to residue retention. This study provides a valuable governance reference and scientific basis for the government to adjust and improve existing policies, including how to stimulate farmers to continue to adopt agricultural waste treatment technologies such as residue retention.

## 1. Introduction

It is common to burn crop residues after harvest in developing countries [1], and China is no exception. As the world’s largest producer of crop residues, China produces almost 20% of the global total crop residues each year [2]. It is estimated that one-quarter of crop residues in China are burned in the fields [3]. Crop residue burning can affect both human health and global climate change. In the past few years, levels of smog caused by crop residues burning throughout China resulted in the deterioration of air quality, creating a significant threat to human health (e.g., lung diseases and premature death) [4,5]. In addition, the open burning of crop residues has been proven to be one of the essential sources of CO_2_ and CH_4_ emissions, leading to global climate change [6].

The sustainable use of crop residues can help reduce the pressure of environmental pollution and alleviate the adverse impacts of climate change [7,8]. Due to the high cost of transporting and collecting crop residues, recycling crop residues used for industrial raw materials and energy production is not a good deal for farmers [9], even though the energy utilization potential of crop residues is huge [10]. Residue retention helps to improve soil quality by increasing soil organic matter, reducing land degradation, and enhancing the ability of soil to absorb carbon [11,12,13,14]. In addition, compared with the use of chemical fertilizers, maintaining and improving soil fertility by crop residue retention is more conducive to preventing soil compaction and erosion, while enhancing soil stability and promoting biodiversity of the surrounding environment [15]. Thus, it is noteworthy that the long-term continuous use of crop residues can effectively increase crop production and farmers’ income [16,17].

To prevent the hazards on human health and the ecological environment caused by the incineration of crop residues, China’s various level governments have issued a series of bans on the incineration of crop residues in the field. For example, the ban in the form of legislation was launched by Hubei province in China to prevent crop residue burning [18]. In addition, the Chinese government also provides supporting policies, including agricultural machinery operation subsidies, agricultural machinery purchase subsidies, and residue retention subsidies to incentivize farmers to adopt residue retention. However, farmers still are reluctant to keep crop residues continuously. According to the China Ministry of Ecology and Environment (MEE), 16,813 fires caused by crop residues occurred from July 2014 to November 2016 in China. Chinese farmers are still burning crop residues, even if they have had the experience of retaining crop residues. Little is known about the reason why farmers are reluctant to keep crop residues continuously [19]. It is necessary and critical to analyze farmers’ continuance intention to crop residue retention and its contributing factors.

In existing studies, measures for the utilization of crop residues and the assessment of the potential of these measures to reduce environmental pollution have been well documented [1,20,21]. The determining factors that are frequently observed are farmer characteristics including age, education, gender, and other factors [22,23]. The psychological factors that affect farmers’ intentions and behavior of crop residue retention have also received widespread attention wide [24,25]. Furthermore, economic factors such as land tenure security and non-economic factors such as media channels and government policies are also important in affecting farmers’ retention of crop residues [26,27]. For example, in their investigation of 670 rural households in China, Gao, et al. [28] found that farmers have a higher probability of crop residue retention of their land than that on their rented land. Jiang, et al. [29] showed that media channels increased the probability that a farmer voluntarily adopts the practice of straw return. Hou, et al. [30] found that increased availability of residue chopper machines and the establishment of demonstration projects promote the adoption of residue retention.

This literature, however, is inconclusive as to which of the explanatory characteristics plays a dominant role in the use of residue retention [31,32]. In particular, there is a lack of convincing explanations for the phenomenon of farmers who initially carried out residue retention, but eventually gave up or intermittently carried out residue retention. Essentially, this reflects the initial acceptance of farmers does not mean that farmers always have a continuous intention to it. However, there is a lack of relevant research on the continuous intention to crop residue retention for farmers.

The main objectives of this research are to explore the key factors that affect farmers’ continuance intention, examine the interaction mechanism between these factors, and evaluate their effects on farmers’ continuance intention to residue retention in China. We contribute to the existing literature in three ways. First, this paper analyzed the main influencing factors of continuance intention to residue retention, using data collected from 542 farmers in the Hubei province of China. To our knowledge, no published studies have analyzed farmers’ continuance intention to residue retention and its influencing factor. Second, this paper estimated the relative importance and interaction mechanism of farmers’ perceived usefulness, confirmation, perceived ease of use, and satisfaction. Third, this study extended the expectation–confirmation model (ECM) adopted in general continuance intention research use context for the applicability of the farmer’s crop residues retention research. This is also the first time that the ECM has been applied to analyze the continuance intentions of farmers. Based on this model, this paper also highlighted the difference between the initial and continued adoption by farmers for the first time, and it demonstrated the potential of ECM to improve our understanding of farmers’ continuance intentions.

Our findings are favorable to comprehend the reasons for crop residue repeated burning in China. It has important policy implications for the government to adjust the existing policies and promote the long-term use of crop residue retention. This paper also provides experience for other developing countries with similar problems. While this paper focuses on the case of China, our empirical findings are relevant for several other countries as China’s agricultural development over the recent decades has certain referential significance for many contemporary developing economies.

The rest of this paper is organized as follows. Theoretical framework and research hypotheses are discussed in Section 2. Data and statistical analysis are presented in Section 3. Section 4 describes the results, and Section 5 reports the discussion. Conclusions are presented in Section 6.

## 2. Theoretical Framework and Hypotheses

### 2.1. Theoretical Framework

In the initial stage of introducing a new service, users’ decision-making is based on the acceptance of the service, which has been proved to fundamentally differ from their continuance-related decisions as time and experience increase [33]. Crop residue retention is not a new service in the initial stage. In most developing countries and regions, including China, the service has been accepted for many years [28]. Since crop residue retention occurs year after year, the background of this study requires consideration of the long-term use of crop residue retention by farmers. Residue retention every year is essentially a continuous behavior of adopting crop residue retention [19]. Farmers’ continuance decision to crop residue retention is similar to information systems users’ continuance decision because both decisions (1) follow an initial (adopt or use) decision, (2) are influenced by the initial use (of information systems or service) experience, and (3) can potentially lead to the ex-post reversal of the initial decision [34,35]. Continuance of crop residue retention often imposes monetary and nonmonetary costs on farmers [1]. Hence, rational farmers, most likely, go through a non-trivial decision process, similar to that of information systems users, before making an informed decision choice. For analyzing the decision-making process of farmers, this paper referred to the theoretical framework of continuance intention of information system service users and employed the ECM to analyze farmers’ crop residue retention intention.

The ECM was developed from Expectation Confirmation Theory (ECT) by Bhattacherjee [34] to evaluate the continuance usage intention of information systems. Compared with the ECM, ECT ignores potential changes in consumers’ expectations after their consumption and the effect of these changes on subsequent cognitive processes. However, ECM pays more attention to the cognition after use, using perceived usefulness instead of expectations after use, and using the two variables of satisfaction and confirmation to describe the information contained in the two variables: the user’s expectation before use and the user’s perceived performance after use [35]. ECM makes up for the shortcomings of the ECT in the study of users’ continuance intention and enhances the ability to explain the continuance intention. It not only includes three aspects of the classical psychological user behavior model (cognition, emotion, and action, intention) but also is consistent with the paradigm of rational behavior theory that belief affects attitude and attitude affects behavior or intention. Potentially ECM can explain information systems continuance decisions better than ECT alone.

The ECM is an important result of previous work on continuance, and its validity has been widely confirmed and used in various continuance contexts over the past decade [34,36,37,38]. As Yuan, et al. [39] extended with perceived ease of use, confirmation, satisfaction, perceived usefulness, perceived task-technology fit, and perceived risk to analyze Chinese users’ continuance intentions of mobile banking. Chong [40] extended ECM with perceived ease of use, perceived enjoyment, trust, and perceived cost and proved that Chinese consumers’ perceived ease of use significantly affected mobile commerce continuance intentions.

The ECM generates three predictors of behavioral intentions toward and the adoption of service: confirmation, perceived usefulness, and satisfaction. Whereas confirmation refers to adopters’ perceptions of the congruence between the expectation of service adoption and its actual performance, and perceived usefulness refers to the degree of expected benefits in using service. Satisfaction is defined as the user’s effect or perception of previous use [35]. The ECM comes from research in the field of information systems, and there may be some limitations when the original constructs contained in ECM are directly used to explain the field of agricultural services. Hong, et al. [41] also confirmed the important influence of situational factors on the effectiveness of information system research and elaborated the necessity of expanding the original model according to specific situational factors. Thus, for the sake of adapting ECM to a specific context (i.e., the continuance of crop residue retention), several extensions are helpful to heighten the explanatory power of the ECM [42].

Considering the uncertainty and long-term nature of residue retention gains and the increasing time input of farmers in higher-paid work such as off-farm employment, farmers may prefer to dispose of the residue quickly and conveniently so that they can devote more time to higher-paid work. For rational farmers, the difficulty of obtaining and using crop residue retention technology should also be an important reason for determining whether their intentions continue or not. Therefore, to better understand the characteristics of crop residue retention farmer experience, combining previous studies, we further incorporated the perceived ease of use into the ECM and extended it in our study.

### 2.2. Development of Hypothesis

#### 2.2.1. Satisfaction

Locke [43] initially proposed that satisfaction is a pleasant and positive emotional state based on job evaluation. According to ECT theory, satisfaction is a kind of emotion, expressed as positive (satisfaction) or negative (dissatisfaction). Satisfaction is defined in ECM-based studies as the user’s effect or perception of previous use [35], and it is generally considered to be an important predictor of the user’s continuance intention. Farmers will be satisfied if perceived residue retention performance exceeded their expectations, which leads to positive action towards continuous usage of crop residue retention. Several studies based on different models provide anecdotal evidence for satisfaction-continuance intention association, such as ECM, the unified theory of use and acceptance of technology (UTAUT), the technology acceptance model (TAM), and their extended or comprehensive model [44,45,46,47]. In that light, we proposed the following hypothesis:

**Hypothesis** **1** **(H1).** *Satisfaction positively affects farmers’ continuance intention to residue retention.*

#### 2.2.2. Perceived Usefulness

Perceived usefulness is defined as users’ subjective probability that information systems use will improve their performance [48], reflects the ex-post expectations of the information system or service [35]. In the field of continuance intention research, perceived usefulness is generally regarded as one of the strongest predictors of continuance intention [34,39,49]. In terms of residue retention, perceived usefulness refers to farmers’ perception of the expected benefits and the improvement of performance. Residue retention can help farmers increase production by improving soil quality and fertility [12,13], and has a certain effect of increasing agricultural income [14]. Besides, it also reduces environmental pollution, which is beneficial to the living environment of farmers. People are always willing to continue to adopt technologies that can benefit them. The better the expected benefits (usefulness) of residue retention perceived by farmers, and the stronger their continuance intention to residue retention. Additionally, for the residue retention that can bring good expected benefits, farmers will naturally have a good feeling and positive emotional evaluation. In other words, farmers think that residue retention is not useful and has no good expected benefits, so their satisfaction is naturally not high. This effect has also been confirmed by many studies [39,49]. Thus, we propose the following two hypotheses:

**Hypothesis** **2** **(H2).** *Perceived usefulness positively influences farmers’ continuance intention to residue retention.*

**Hypothesis** **3** **(H3).** *Perceived usefulness positively influences farmers’ satisfaction.*

#### 2.2.3. Perceived Ease of Use

Perceived ease of use is defined as the degree to which using a product or service would be effortless [48], it shows the time or effort that farmers need to pay to adopt residue retention. Many studies have shown that perceived ease of use positively affects continuance, both directly and indirectly, via its effect on satisfaction [37,50]. Compared with crop residues burning in the open air, residue retention is a little more complicated. If this “complexity” is beyond farmers’ acceptance, residue retention will not be an easy task for farmers, which will not be conducive to their continuance intention to residue retention. Besides, easy-to-use residue retention will reduce the energy and time spent by farmers, make it easier to get the favor of farmers and improve their satisfaction. Therefore, the following hypotheses are proposed:

**Hypothesis** **4** **(H4).** *Perceived ease of use positively affects farmers’ continuance intention to residue retention.*

**Hypothesis** **5** **(H5).** *Perceived ease of use (time, effort) positively influences farmers’ satisfaction.*

#### 2.2.4. Confirmation

Perceived confirmation represents consumers’ subjective post-only rating of the same discrepancy, either at overall product or service level or individual attribute level [51,52]. Kim and Malhotra [53] believe that users’ early evaluation will have an impact on their late evaluation in the setting of continuance, because the knowledge from their own experience is the most critical information basis for users to make decisions, and previous experience can help individuals evaluate the results more clearly and forcefully [54]. The confirmation is a proper variable to capture the relevant information of previous experience [35]. In this study, we defined confirmation of farmers as the gap between the expected value of residue retention before receiving the service and the value experienced after using the service. To simplify, Confirmation represents farmers’ perception of the congruence between the expectation of residue retention and its actual performance. There may be inconsistencies or gaps between farmers’ expectation cognition and actual performance, cognition, which will lead to cognitive imbalance and psychological tension [35]. According to the cognitive dissonance theory, rational users can try to make up for this disharmony by adjusting their usefulness perceptions and ease of use perceptions, to be more consistent with reality. In other words, the confirmation will enhance users’ perceived usefulness and perceived ease of use, while disconfirmation weakens this perception.

Previous studies have confirmed that confirmation significantly affects users’ perceived usefulness and their perceived ease of use [37,39,49,50]. Additionally, confirmation positively influences satisfaction [35,46,55]. In this study, the confirmation will lead to the farmer’s satisfaction once their experience of residue retention or exceeds their initial expectation. In contrast, if the actual use of residue retention fails to meet the initial expectation, farmers will be dissatisfied. Hence, the following hypotheses were proposed based on the above analysis:

**Hypothesis** **6** **(H6).** *Confirmation positively affects the perceived usefulness of residue retention.*

**Hypothesis** **7** **(H7).** *Confirmation positively affects the perceived ease of use of residue retention.*

**Hypothesis** **8** **(H8).** *Confirmation positively affects farmers’ satisfaction with residue retention.*

**Figure 1:**
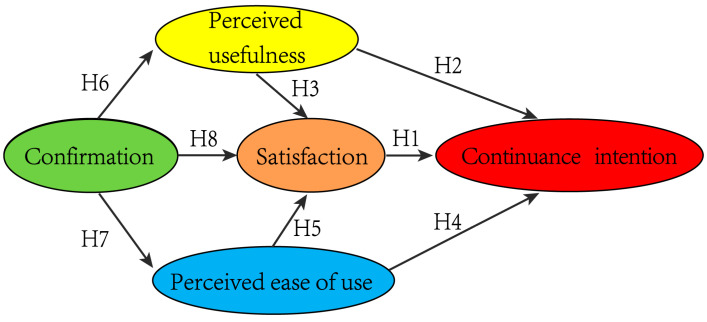
The theoretical model of farmers’ continuous intention to residue retention.

According to previous literature and the proposed hypotheses, the research model presents the hypotheses paths in Figure 1.

## 3. Data and Statistical Analysis

### 3.1. Study Area

Hubei province is the leading grain production area of China, which is covered by 4645 million hectares of farm sizes. Based on the local meteorological data, except for high mountain regions, most of the province belonged to humid subtropical monsoon climate, with annual precipitation ranges between 800 and 1600 mm. Hubei is an interesting example to study farmers’ continuance intention of crop residue retention for two reasons. First, the largest grain crop of the province was rice, with an annual planting area of about 2335 million hectares and a total output of about 19 million tons. The area and total production of Hubei are ranked sixth and fifth in China all year round, and it is one of the main rice-producing provinces in China. Hubei Province produces a huge amount of straw every year, the disposal and utilization of crop residues is a common problem faced by most farmers in Hubei Province. Second, fertilizer utilization represented by residue retention is the main treatment method of crop residue in Hubei, which is very familiar to most farmers.

### 3.2. Data

**Figure 2:**
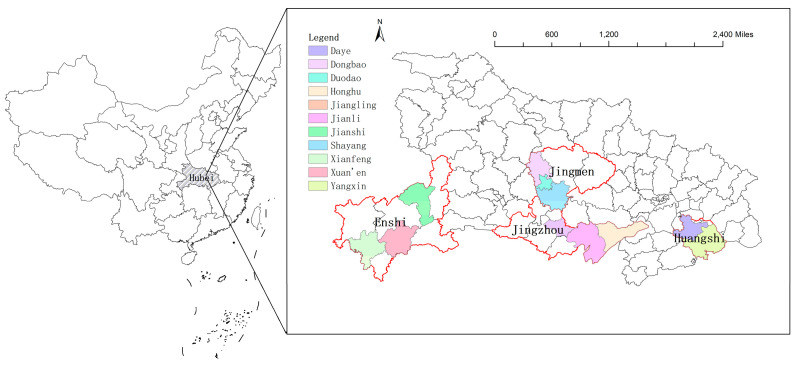
Description of study areas.

This household questionnaire survey was conducted from July to September 2018 by a multistage random sampling procedure. To further explore farmers’ intention to residue retention, we conducted a household survey in 11 counties of Enshi, Jingmen, Jingzhou, and Huangshi in Hubei province from July to September 2018, that is, Xuanen, Jianshi, Xianfeng, Shayang, Duodao, Dongbao, Jianli, Honghu, Jiangling, Yangxin, and Daye (Figure 2). The research group randomly selected 2–3 counties in each city, 1–2 towns in each county, and 2–3 villages in each town. According to the list obtained from the village committee, we also randomly selected respondents from 15 to 25 households in the sample villages to conduct a questionnaire survey. The survey took the form of a face-to-face interview between an investigator and an interviewed farmer. The investigators were full-time master’s or doctoral students at Huazhong Agricultural University and received unified skills training in advance of the interviews. A total of 724 farmers were surveyed. Finally, after eliminating the invalid samples such as incomplete or inconsistent key information, we collected data from a total of 542 samples.

### 3.3. Measurement of Key Variables

The questionnaire included five parts: (1) Farmers’ demographic and socioeconomic information; (2) farmers’ basic understanding of agricultural wastes such as crop residue; (3) the promotion of technical services such as residue retention; (4) The service needs of residue retention; (5) farmers’ response to the problems related to residue retention.

We designed a scale to obtain micro-data of farmers’ continuance intention to residue retention. Four experts and six graduate students from relevant research fields were invited to conduct discussions on each item to ensure the content validity of the questionnaire and the measurement accuracy of the scale. Based on the feedback of the pre-survey subjects, we modified the questionnaire to comply with the rural reality and be easily understood by farmers.

In particular, the design of the scale of farmers’ perceived usefulness (4 items) and farmers’ perceived ease of use (4 items) referred to the research results of Premkumar [50] and Bhattacherjee [34], while the confirmation (4 items) was improved according to the scale developed by Thong, Hong, and Tam [37]. Satisfaction (3 items) was drawn from the research design of Hsu, et al. [56] and Kim, et al. [57]. Continuance intention (3 items) scale was mainly developed by [35]. The formal scale consisted of 18 items, all of which were measured with a five-point Likert scale from 1 = “totally disagree” to 5 = “very agree”. The formal questionnaire formed after the pre-survey included five latent variables and eighteen items, summarized in Table 1.

### 3.4. Statistical Analysis

Structural equation model (SEM) analysis is conducted to estimate the conformation of the questionnaire data and the hypothesized model by using SPSS (version 19.0) (IBM, Armonk, NY, USA) and AMOS (version 22.0) (IBM, Armonk, NY, USA). SEM is an effective empirical method for the comprehensive evaluation of theoretical models, especially since the model can effectively identify the relationship between multiple cubic variables that are difficult to directly measure [58]. In this study, we implemented the maximum likelihood approach as the model estimation method.

The reliability and validity were firstly tested. The reliability test was measured by Cronbach’s α coefficient and combination reliability. According to Hair, et al. [59], Cronbach’s α value can be less than 0.7, at least above 0.5, and indicates bad reliability if it is less than 0.35. Devillis (2003) believes that a Cronbach’s α value between 0.60 and 0.70 indicates good reliability, while a value above 0.70 indicates great reliability. The confirmatory factor analysis was used to measure the validity of the scale in this paper. Kaiser-Meyer-Olkin (KMO) and Bartlett’s test of sphericity are universal methods to measure sampling suitability, adequacy, and factorability. The sampling is not adequate if a KMO value of less than 0.6 [59].

Besides, a model fitness test is necessary to ensure the degree that the theoretical model accords with the actual data. The overall fitness test index of the structural equation model mainly includes the absolute fitness index, value-added fitness index, and reduced fitness index. The goodness-of-fit indices of the model are judged according to the conventional rules of thumb of Joe, et al. [60].

Finally, confirmation may affect farmer’s continuance intention to residue retention through satisfaction, perceived usefulness, perceived ease of use. Therefore, mediating effect analysis needs to be conducted in this paper. According to the research of Taylor, et al. [61] and Hayes and Scharkow [62], this paper uses the Bias-Corrected Bootstrap program for mediating effect tests.

## 4. Results

### 4.1. Farmers’ Demographic Profile

As shown in Table 2, the respondents interviewed are mainly male, accounting for 87.45% of the total number of samples. The age of the surveyed farmers is on the high side (51 and above), accounting for 63.84%. Most of the farmers have a junior high school education with a percent of 55.17%. Only 2.77% of the interviewed farmers have a college education or above, and the education level of the interviewed farmers is generally low. Some farmers (33.76%) also have part-time jobs in addition to agriculture. The farmers interviewed have rich experience in farming; 52.95% of the surveyed farmers have more than 30 years of farming experience. 37.27% of the surveyed farmers’ annual agricultural income accounts for more than 50% of the total household income. 55.54% of the surveyed farmers have a planting scale of less than 0.67 ha. On the whole, agricultural income is an important source of income for the surveyed farmers, and the scale of agricultural production is still mainly small and medium-sized.

### 4.2. Reliability and Validity Test

Results of the reliability test are presented in Table 3, the Cronbach’s α coefficients of each latent variable are mostly ranging from 0.724 to 0.940, and the combined reliability are mostly between 0.730 and 0.827, indicating that the internal consistency of the measurement index of the scale is great and the reliability is high.

The results of the factor analysis applicability test in Table 3 show that the Kaiser-Meyer-Olkin (KMO) values of the scale are all higher than 0.6, and the significant *p*-values of Bartlett’s spherical test are higher than 0.05, indicating that the measurement indexes of the scale have a high correlation and are suitable for confirmatory factor analysis. The results show that all standardized factor loads exceed 0.5 and significant at the 0.05 level, indicating that the observed variables have good discriminant validity. At the same time, the parameter estimation between the measurement item and the potential matching structure is statistically significant at a 1% level, indicating that all measurement items are strong enough to explain their corresponding potential structure. On the whole, the reliability and validity of the measurement model have been confirmed.

In addition, the lower part of Table 4 presents the goodness-of-fit. All of these test indexes reached the recommended value, which indicates that the fitness of the measurement model is reasonable.

### 4.3. Hypothesis Test

**Figure 3:**
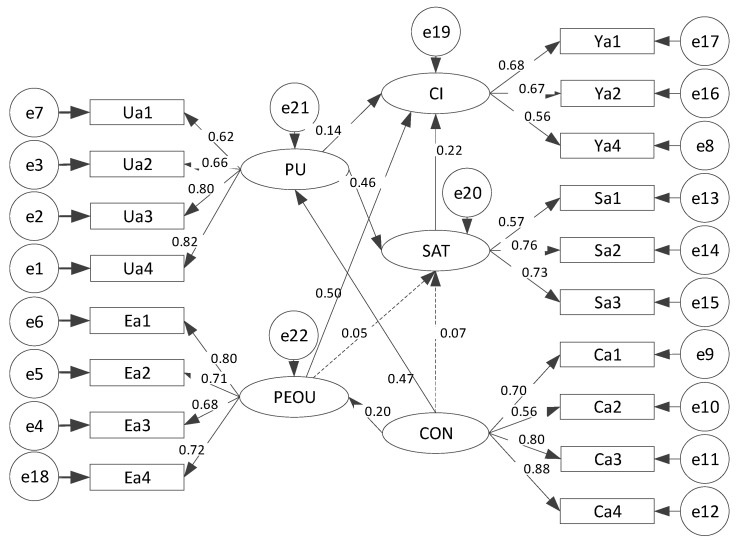
Standard structural load and path factor based on the final structural model of the extended ECM. The circle represents the error term, the square represents the tested term, the ellipse represents the potential structure, and the arrow represents the dependency. The solid arrow indicates a significant impact, while the dashed arrow indicates that the effect is not substantial.

Table 5 presents the estimation result of the structural equation model. The graphical representation of Table 5 is shown in Figure 3.

The standardized path coefficient of the influence of the farmers’ satisfaction on their continuance intention to residue retention is 0.216, and it was significant at the statistical level of 0.05, thus verifying hypothesis H1. This indicates that the more satisfied the farmers are with residue retention, the stronger their continuance intention to residue retention. The path from the PU to CI is significant (standardized path coefficient = 0.141; *p* < 0.1). Thus, hypothesis H2 was verified, indicating that the stronger the farmers’ perceived usefulness, the stronger their continuance intention to residue retention. The path from the PU to SAT is significant (standardized path coefficient = 0.464; *p* < 0.01), so hypothesis H3 was verified, indicating that the more influential the farmers’ perceived usefulness is, the higher the satisfaction with the assistance of residue retention.

The statistically significant path from PEOU to CI (standardized path coefficient = 0.505, *p* < 0.01) verifies hypothesis H4, indicating that the more influential the farmers’ PEOU, the stronger their continuance intention to residue retention. The path from PEOU to SAT is not significant (standardized path coefficient = 0.047; *p* = 0.362), this hypothesis H5 was not verified, indicating that farmers’ PEOU does not significantly influence farmers’ SAT of residue retention.

The standardized path coefficients of farmers’ confirmation impact of residue retention on their PU and PEOU are 0.475 and 0.204, respectively, and significantly verified the hypotheses H6 and H7. The results show that the higher the positive value of the confirmation, the stronger the farmers’ perception of the usefulness and ease of use of the residue retention. The standardized coefficient of the influence of farmers’ confirmation on the satisfaction of residue retention is not statistically significant (standardized path coefficient = 0.073; *p* = 0.217), the results reject hypotheses H8.

### 4.4. The Results of Mediating Effect Analysis

To test the mediating effect that satisfaction, perceived usefulness, and perceived ease of use may play in confirmation and continuance intentions, this paper uses the Bias-Corrected Bootstrap program for mediating effect test, takes 5000 Bootstrap samples from the original data utilizing repeated random sampling, generates an approximate sampling distribution, and selects the confidence interval of 95% confidence to estimate. The results of mediating effect analysis are shown in Table 6. The results show that PU and PEOU play a complete intermediary role between CON and CI. In other words, the CON of farmers can also directly affect their CI through PU or PEOU. PEOU and PU play a complete dual intermediary role between the CON and farmers’ CI. In other words, the confirmation of farmers can also affect their satisfaction through perceived usefulness and ultimately transform their continuance intention to residue retention.

The paths of farmers’ continuance intention to residue retention come from three ways: first, through the influence of CON on PU; second, CON affects PEOU and then affects CI; third, CON affects farmers’ continuance intention to residue retention through the dual intermediary role of PU and SAT. Furthermore, based on estimating the indirect, direct, and total effect of the four latent variables, this paper calculates the influential effects of each path, respectively. Thus, it can directly show the influence degree of the latent variables of different paths on farmers’ continuance intention to residue retention. The results show that the influence effect of path 1 (CON→PU→CI) is 0.067, which means that the change of confirmation of each standard deviation in this path will lead to a shift in farmers’ continuance intention 0.067 units. The influence effect of path 2 (CON→PEOU→CI) is 0.103, which means that for every standard unit of conformation change, the change of farmers’ continuance intention to residue retention is 0.103 units. The influence effect of path 3 (CON→PU→SAT→CI) is 0.048, indicating that the change of the confirmation of each standard deviation results in the shift of farmers’ continuance intention to residue retention is 0.048 units. The effective level of path 2 is the highest among the three influence paths, which is significantly higher than that of path 1 and path 2.

## 5. Discussion

Collectively, our findings strongly support the applicability of the extended expectation confirmation model of information system continuance in predicting the intention of continuing usage of agricultural waste by Chinese farmers. Our study reveals that farmers’ continuance intention to residue retention is determined by satisfaction, perceived usefulness, perceived ease of use, and confirmation. It can be concluded that three (satisfaction, perceived usefulness, and perceived ease of use) of the four variables emerged as statistically significant antecedents of continuance intention to residue retention. Wherein satisfaction positively and significantly affects farmers’ continuance intention. This relationship has been confirmed in a wide range of other contexts in continuance intention to mobile technologies and services [39,49]. Satisfaction plays a mediation role between perceived usefulness and farmers’ continuance intention, and perceived usefulness has also been proved to be a crucial indicator affecting farmers’ continuance intention in our sample. Confirmation simultaneously plays a vital predictor role in influencing satisfaction and perceived usefulness positively. This finding is consistent with the main findings in the literature [39]. Therefore, improving farmers’ satisfaction by providing a high quality of service to meet farmers’ requirements, and sustainably maintaining future development of residue retention by satisfying farmers’ service and mental expectations, are essential and appropriate approaches for residue retention providers to retain farmers. Furthermore, the stakeholders of residue retention should guarantee the performance and usability of residue retention accommodation to farmers’ expectancy, and improve users’ experience based on farmers’ requirements and different situations to increase farmers’ satisfaction and maintain their continuance intention. For instance, demonstration projects, providing a platform for collecting and solving these problems, should be set up to allow farmers to fully express their requirements and problems [30].

According to the results of path analysis, perceived ease of use ranks first in influencing farmers’ continuance intention, which is significantly higher than the path of perceived usefulness and the path of satisfaction. The results suggest that farmers’ continuance intention to residue retention was mainly motivated by a desire to spend the least time and energy to crop residue disposal. Our finding is contrary to the previous findings that satisfaction has the most substantial effect on continuance intention [44,46]. Whether residue retention is useful or can satisfy farmers is not the primary factor for farmers to consider, although it could also benefit farmers by improving soil quality and fertility [12,13,14]. One possible explanation is that crop residues are a waste rather than a resource for farmers. Farmers are more willing to spend their time and energy on non-farm farming to obtain higher economic benefits [63,64]. Therefore, improving the ease of use when designing residue retention-related machinery should be considered as an effective way to promote crop residue retention. At the same time, residue retention can reduce the time and effort input of crop residue treatment, which should also be the focus of publicity and promotion to farmers, thereby enhancing their continuance intention to residue retention. For this purpose, it is necessary to make full use of television, radio, news media, and so on to the propaganda and report on this advantage. In addition, residue retention machinery production and sales enterprises and social service organizations also need to be encouraged to carry out relevant training, to improve further farmers’ awareness of the ease of use of residue retention.

Notably, confirmation significantly impacts both perceived usefulness and perceived ease of use, and the effect of confirmation on perceived usefulness is relatively high. The findings are consistent with existing studies [37,39,49,50]. The confirmation reflects farmers’ expectations formulated by their previous experience of residue retention. In their investigation of an online cross-sectional survey, Ray, et al. [65] found experience directly impacts users’ intention to FDA’s. The findings of this paper supplement their findings with mediating variables perceived ease of use, which explains the technological effects of confirmation on farmers’ continuance intention to residue retention. Thus, residue retention service or product providers should improve the efficiency and effortlessness of residue retention, use to increase farmers’ continuance intention to residue retention.

Although we have made cautious suggestions to promote crop residue retention, it does not mean that all regions are suitable for this service. The implementation of crop residue retention needs to be coordinated with local climate, land topography, infrastructure, and other factors. Therefore, in suitable areas, it is suggested to increase the promotion of crop residue retention. In other areas, we hope that the relevant research institutions can develop a more suitable crop residue treatment model for the local reality. In a word, only the services adapted to local conditions can be long-term accepted and welcomed by farmers, and only such services can realize the sustainable utilization of farmers.

## 6. Conclusions

Crop residue burning in the fields is particularly prominent and prevalent in China. Burning crop residue in the field can release harmful pollutants and greenhouse gases. Promoting crop residue retention to replace residue burning is very effective in improving air quality and contributing to the mitigation of global climate change. In this paper, we analyze the main influencing factors of farmers’ continuance intention, estimate the relative importance and interaction mechanism of farmers’ perceived usefulness, confirmation, perceived ease of use, and satisfaction and evaluate their effects on farmers’ continuance intention to residue retention in China, using an extended ECM.

The empirical results reveal the main influencing factors of continuance intention to residue retention. The findings show that farmers’ perceived usefulness, perceived ease of use, confirmation, and satisfaction are the key factors that affect their intention to residue retention. Among them, perceived ease of use has the greatest impact. Farmers’ perceived usefulness of residue retention is an intermediary or a bridge that mediates confirmation and satisfaction. Perceived usefulness and perceived ease of use are capable of exerting an intermediary effect on the relationship between confirmation and farmers’ continuance intention.

Our results may not be directly generalized to other parts of China due to the substantial regional differences in crop residue treatment requirements across China. However, while our results only apply to Hubei province in China, they do show the factors and paths influencing farmers’ continuance intention to crop residue retention. At least in terms of Hubei province, our findings provide a valuable governance reference and scientific basis for the policymakers to adjust existing policies, including how to stimulate farmers to continue to adopt agricultural waste treatment technologies.

There are still some limitations in this paper. We mainly focus on the farmers’ continuance intention in this study, rather than their actual continuance behavior. There may be some differences between intention and action [66], and future research can use persistent behavior to test the extended ECM. On the other hand, this paper only validates the applicability of the extended ECM in explaining farmers’ continuous use of residue retention. Further research may try to add some predictive factors to improve the predictability of the model for farmers’ continuance intention and action of agricultural waste, then assess the gap between their intention and act in the treatment of agricultural waste such as crop residue to reduce air pollutants and greenhouse gases. Notably, crop residue is only one type of agricultural waste. In terms of crop types, this paper mainly focused on grain residue retention. Future research can build on the model of this study and extend the conclusions to different agricultural planting residues and other agricultural waste.

## Figures and Tables

**Table 1:** Latent variables measurement and descriptive statistics.

Latent Variables	Item	Statement	Mean	Standard Deviation
Perceived usefulness	Ua1	Overall, residue retention is conducive to the control and amelioration of air pollution.	4.177	0.850
	Ua2	Residue retention is beneficial to increase the farmland’s yield of the family.	3.699	0.856
	Ua3	Residue retention is conducive to promoting the development and progress of society.	4.044	0.751
	Ua4	Residue retention is beneficial to improve the living environment.	4.146	0.734
Perceived ease of use	Ea1	The skilled use of residue retention is easy for me.	3.435	1.147
	Ea2	Now I fully remember the operation process of residue retention.	3.714	1.073
	Ea3	Accomplishing a specific goal by residue retention is easy for me.	3.812	1.102
	Ea4	Residue retention is effortless for me.	3.275	1.154
Confirmation	Ca1	After using residue retention, I found that the productive input was better than expected.	3.576	0.743
	Ca2	The improvement of cultivated land with residue retention is more considerable than expected.	3.120	0.980
	Ca3	After residue retention, agricultural income is better than expected.	3.727	0.759
	Ca4	My expectations for residue retention have been realized.	3.648	0.759
Satisfaction	Sa1	The service of residue retention satisfied me.	3.470	0.913
	Sa2	The work of the residue retention provider satisfied me.	3.255	1.061
	Sa3	Generally speaking, I am satisfied with the service level and the effect of residue retention.	3.363	1.028
Continuance intention	Ya1	I intend to continue to adopt residue retention.	3.690	1.268
	Ya2	I intend to continue to adopt residue retention instead of related alternative technologies and services.	3.961	1.125
	Ya3	I will continue to residue retention for the rest of my life.	4.277	0.910

**Table 2:** Socio-economic characteristics of surveyed farmers.

Index	Item	Sample Size	Proportion (%)	Index	Item	Sample Size	Proportion (%)
Gender	Male	474	87.45	Farming experience	≤10 year	41	7.56
	Female	68	12.55		11~20 year	69	12.73
Age	≤30	8	1.48		21~30 year	145	26.75
	31~40	35	6.46		≥31 year	287	52.95
	41~50	153	28.23	The proportion of annual household, agricultural income	≤25%	210	38.74
	51~60	188	34.69		25%~50%	130	23.99
	≥61	158	29.15		50%~75%	71	13.10
Educational level	Elementary/below	124	22.88		≥75%	131	24.17
	Junior high	299	55.17	Planting scale	≤0.33 ha	166	30.63
	High/ vocational	104	19.19		0.33~0.67 ha	135	24.91
	College/above	15	2.77		0.67~1.00 ha	97	17.90
Part-time job	Yes	183	33.76		1.00~1.33 ha	77	14.21
	No	359	66.24		≥1.33 ha	67	12.36

**Table 3:** Result of reliability and validity test.

Construct	Item	Factor Loadings	Cronbach’s α	KMO	CR	AVE
Perceived usefulness (PU)	Ua1	0.620	0.802	0.774	0.819	0.534
	Ua2	0.655				
	Ua3	0.804				
	Ua4	0.822				
Perceived ease of use (PEOU)	Ea1	0.798	0.815	0.771	0.817	0.529
	Ea2	0.713				
	Ea3	0.675				
	Ea4	0.718				
Confirmation (CON)	Ca1	0.698	0.805	0.773	0.827	0.552
	Ca2	0.555				
	Ca3	0.795				
	Ca4	0.883				
Satisfaction (SAT)	Sa1	0.572	0.724	0.653	0.730	0.478
	Sa2	0.759				
	Sa3	0.728				
Continuance intention (CI)	Ya1	0.678	0.666	0.652	0.670	0.405
	Ya2	0.667				
	Ya3	0.558				

**Table 4:** Model fitness test.

Inspection Index		Recommend Value	Index Result	Test Results
Absolute fitness index	CMIN/DF	[2,5]	2.379	Fit
	RMSEA	<0.100	0.050	Fit
	GFI	>0.800	0.942	Fit
	AGFI	>0.800	0.922	Fit
Value-added fitness index	NFI	>0.800	0.914	Fit
	RFI	>0.800	0.897	Fit
	IFI	>0.800	0.948	Fit
	TLI	>0.800	0.937	Fit
	CFI	>0.800	0.948	Fit
Parsimonious fitness index	PGFI	>0.500	0.700	Fit
	PNFI	>0.500	0.759	Fit
	PCFI	>0.500	0.787	Fit

Note: AGFI, adjusted goodness-of-fit index; CFI, comparative fit index; CMIN/DF, chi-squared fit statistic; GFI, the goodness-of-fit index; IFI, incremental fit index; NFI, normed fit index; PGFI, parsimonious goodness-of-fit index; PNFI, parsimonious normal-fit index; RMSEA, root mean square error of approximation; RFI, relative fit index; TFL, Tucker-Lewis index; PCFI, parsimonious comparative fit index.

**Table 5:** Model hypothesis test and path coefficient.

Hypothesis	Path	Coef.	S.E.	C.R.	*p*	Std. Coef.
H1	SAT→CI	0.210	0.067	3.122	0.002 **	0.216
H2	PU→CI	0.119	0.055	2.166	0.030 *	0.141
H3	PU→SAT	0.402	0.062	6.518	***	0.464
H4	PEOU→CI	0.344	0.046	7.403	***	0.505
H5	PEOU→SAT	0.033	0.036	0.911	0.362	0.047
H6	CON→PU	0.552	0.061	9.111	***	0.475
H7	CON→PEOU	0.292	0.074	3.925	***	0.204
H8	CON→SAT	0.074	0.060	1.234	0.217	0.073

*** *p* < 0.01, ** *p* < 0.05, * *p* < 0.1.

**Table 6:** Test results of the mediating effect based on the Bootstrap method.

Path	Direct Effect	Bias-Corrected Confidence Intervals		Indirect Effect	Bias-Corrected Confidence Intervals		Total Effect
		Lower Bounds	Upper Bounds		Lower Bounds	Upper Bounds	
CON→PU	0.475 **	0.436	0.681	--	--	--	0.475
CON→PEOU	0.204 **	0.160	0.442	--	--	--	0.204
CON→SAT	0.073	-0.05	0.209	0.230 **	0.153	0.346	0.303
CON→CI	--	--	--	0.236 **	0.151	0.334	0.236
PU→SAT	0.464 **	0.272	0.560	--	--	--	0.464
PEOU→SAT	0.047	-0.035	0.108	--	--	--	0.047
PU→CI	0.141 **	0.005	0.259	0.100 **	0.034	0.160	0.242
PEOU→CI	0.505 **	0.251	0.455	0.010	-0.007	0.027	0.515
SAT→CI	0.216 **	0.086	0.364	--	--	--	0.216

Note: ** *p* < 0.05

## Data Availability

The data presented in this study are available on request from the first author.
